# A GRIA2 and PAX8-positive renal solitary fibrous tumor with NAB2-STAT6 gene fusion

**DOI:** 10.1186/s13000-015-0386-x

**Published:** 2015-09-04

**Authors:** Osamu Ichiyanagi, Hiromi Ito, Satoshi Takai, Sei Naito, Tomoyuki Kato, Akira Nagaoka, Mitsunori Yamakawa

**Affiliations:** Department of Urology, Yamagata University Faculty of Medicine, 2-2-2, Iida-Nishi, Yamagata City, Yamagata prefecture 990-9585 Japan; Department of Pathological Diagnostics, Yamagata University Faculty of Medicine, 2-2-2, Iida-Nishi, Yamagata City, Yamagata prefecture 990-9585 Japan

## Abstract

Solitary fibrous tumor (SFT) is a rare neoplasm composed of mesenchymal-derived spindle cells. Although SFT occurs anywhere in the body, they most frequently affects the thoracic region. Here, we reported an extremely rare case of an extrathoracic SFT occurring primarily in the kidney. To our knowledge, little information has been described on the immunohistochemistry (IHC) and genetics of renal SFT.

A 41-year old Japanese female came to our hospital for further examination of a left kidney mass detected incidentally with ultrasound. Extensive investigation of the tumor, including physical, laboratory, and image examinations led to a clinical diagnosis of renal cancer (cT1aN0M0), which were in most parts imbedded in the lower polar parenchyma. The patient underwent laparoscopic radical nephrectomy. The mass was diagnosed pathologically as SFT originating from the kidney, but not as renal carcinoma. Microscopically, the tumor was composed of spindle-shape cells distributed variably in dense collagenous stroma and had a focal hemangiopericytomatous staghorn-like vascular pattern. Mitotic figures, atypical structures, necrosis and hemorrhage were not identified. No adjuvant therapies were given postoperatively. The patient has been free of tumor recurrence for 25 months since the surgery. IHC revealed that the tumor diffusely expressed CD34, CD99, Bcl2, PAX8, NAB2, STAT6, and GRIA2. The tumor stained negatively for desmin, S-100, c-Kit, CK-AE1/AE3, CDK4 and MDM2. A NAB2-SATA6 gene fusion was detected in tumor cells by reverse transcription-polymerase chain reaction, direct sequencing, and an *in situ* proximity ligation brightfield assay. The gene fusion occurred as an 831 bp truncation of exon 2 in *NAB2* connected to the beginning of exon 3 in *STAT6*. We have reported a case of GRIA2 and PAX8-positive SFT occurring primarily in the kidney with such *NAB2*-*STAT6* gene fusion for the first time. Diffuse expression of PAX8 in the tumor might present with a renal origin. Reportedly, benign histology of SFT cannot necessarily predict favorable clinical prognosis. Genetic alterations recently identified in SFT could possibly refer to risk stratification for tumor recurrence. However, malignant preponderance of extrathoracic SFT over thoracic SFT remains unexplained so far. Long-term follow-up after surgery should be performed in the present case.

**Electronic supplementary material:**

The online version of this article (doi:10.1186/s13000-015-0386-x) contains supplementary material, which is available to authorized users.

## Background

A solitary fibrous tumor (SFT) is a rare spindle cell neoplasm originating from mesenchymal cells [1]. It was first depicted in the pleura of the lung by Klemperer et al. [2] in 1931. To date, SFTs have been reported in various locations other than the pleura, including the meninges, orbit, neck, nose, paranasal cavity, thyroid, mediastinum, adrenal gland, liver, pancreas, retroperitoneum, spermatic cord, skin, extremities, uterine cervix, prostate, urinary bladder and kidney [3–5]. Among these affected sites, renal SFT, first reported by Gelb et al. [6] in 1996, is very rare. Approximately only 50 cases have been described in the literature [7]. Microscopy and immunohistochemistry (IHC) of surgically resected or biopsied tumor specimens is essential for SFT diagnosis, as imaging modalities, including ultrasonography (USG), computed tomography (CT) and magnetic resonance imaging are unable to definitively differentiate SFT from renal cell carcinoma (RCC) in most cases [8, 7]. Although SFT is generally regarded as a benign tumor, up to 10 % of extrapleural SFTs exhibit malignant behavior, such invasion, recurrence, or distant metastasis [9–11]. SFT in the kidney is mostly benign but has malignant potential. Recently, there has been an increase in the number of reports of malignant SFT, including metastasis to lymph nodes [12], lung [10, 13, 14], liver [13, 14], pancreas [11] and bone [15, 16], local recurrence [17, 3], peritoneal implantation [3], and invasion to renal vein [18]. Microscopically, the diagnostic criteria for malignant SFT is the presence of increased cellularity with crowded and overlapping nuclei, pleomorphism, necrosis and hemorrhage, increased mitotic activity more than four mitoses per ten high power fields (HPFs) [19, 8, 7]. However, some SFT patients without the malignant features in microscopy presented with distant metastasis [13, 14] and local invasion [18], suggesting that non-malignant histology in SFT could not necessarily predict benign behavior and favorable prognosis in clinical practice.

Recently, *NAB2-STAT6* gene fusions have been reported in SFT [20–22]. Variations of *NAB2-STAT6* gene fusions could affect the clinical features, histology, and prognosis of SFT [23, 24]. IHC of the diagnostic markers STAT6 and GRIA2 would be important in differentiating SFT from other soft tissue tumors [4, 5, 25, 26]. To our knowledge, however, genetic and IHC analyses have only been performed using tissue specimens from SFTs in areas of the body other than the kidney, possibly because renal SFT is extremely rare. In the present paper, we report a patient who underwent radical nephrectomy for treatment of cT1a RCC, which was postoperatively diagnosed by pathology as renal SFT with non-malignant nature. *NAB2-STAT6* gene fusions and IHC with anti-NAB2, -STAT6, -GRIA2 and -PAX8 antibodies were also examined.

## Case presentation

### Clinical summary

A 41-year-old Japanese woman visited our institution for further examination of a left renal mass with a diameter of 3.3 cm detected incidentally by abdominal USG at a municipal health workup for citizens 2 months prior. She had been asymptomatic without any history of local discomfort, hematuria, fever, sweat, or weight loss. Her past and family histories were unremarkable, except that she had taken oral contraceptives regularly for 10 years. She was a never-smoker.

**Fig. 1:**
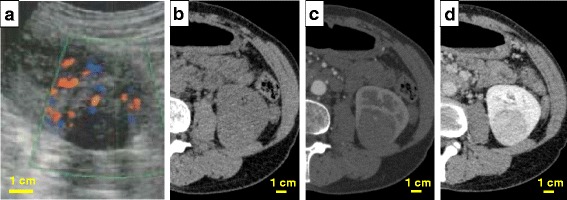
Clinical images of the left renal tumor. A mass with clear margin and mostly endophylic growth was detected in the lower and posterior part of the left kidney. **a** The tumor presented with a non-cystic mass of a heterogeneous nature in ultrasound. Color Doppler imaging showed hypervascularity in the tumor. **b**, **c**, **d**) Dynamic computed tomography of the left renal tumor. **b** Image before administration of the contrast medium. **c** Early phase of enhanced computed tomography. A blood vessel was visualized in the tumor. **d** The tumor showed earlier washout of enhancement than the adjacent normal renal parenchyma

In a physical examination, the patient was normotensive and not obese, with a height of 156.3 cm and weight of 49.5 kg. Laboratory tests, including blood cell counts, biochemistry, C-reactive protein, and urinalysis, showed unremarkable results. A solid, well-demarcated, and heterogeneous mass lesion was observed in the lower pole of the left kidney by USG, accompanied by arterial signals of blood flow within the tumor on color-Doppler ultrasound (Fig. 1a). Plain CT of the tumor showed a faintly higher attenuation than the outer parenchyma of the left kidney (Fig. 1b). The tumor, mostly endophylic in the renal parenchyma, measured 38.0 × 37.5 × 33.6 mm in size in contrast-enhanced CT. Intratumoral serpentine blood vessels were noted in the hypoenhanced tumor tissue in the arterial phase of dynamic CT (Fig. 1c). Subsequently well-enhanced, the tumor showed early washout of contrast media in the delayed phase (Fig. 1d). There was no evidence of local invasion to the adjacent tissues or metastasis to local lymph nodes or distant organs in CT of the chest, abdomen, and pelvis.

Preoperatively, the patient was diagnosed with papillary type I or chromophobe RCC at clinical stage I (cT1aN0M0). The R.E.N.A.L. nephrometry score was 1 + 2 + 3 + p + 1 = 7p for R, E, N, A and L points, respectively, showing moderate complexity of the tumor mass [27, 28]. Endophylic lesions in the kidney are generally considered to be more challenging to surgical resection than exophylic ones [28]. The patient consequently underwent laparoscopic left radical nephrectomy. The postoperative course was not eventful. No adjuvant treatment was administered after surgery and the patient has been free of tumor recurrence or metastasis for 25 months.

### Pathological findings of the surgical specimen

**Fig. 2:**
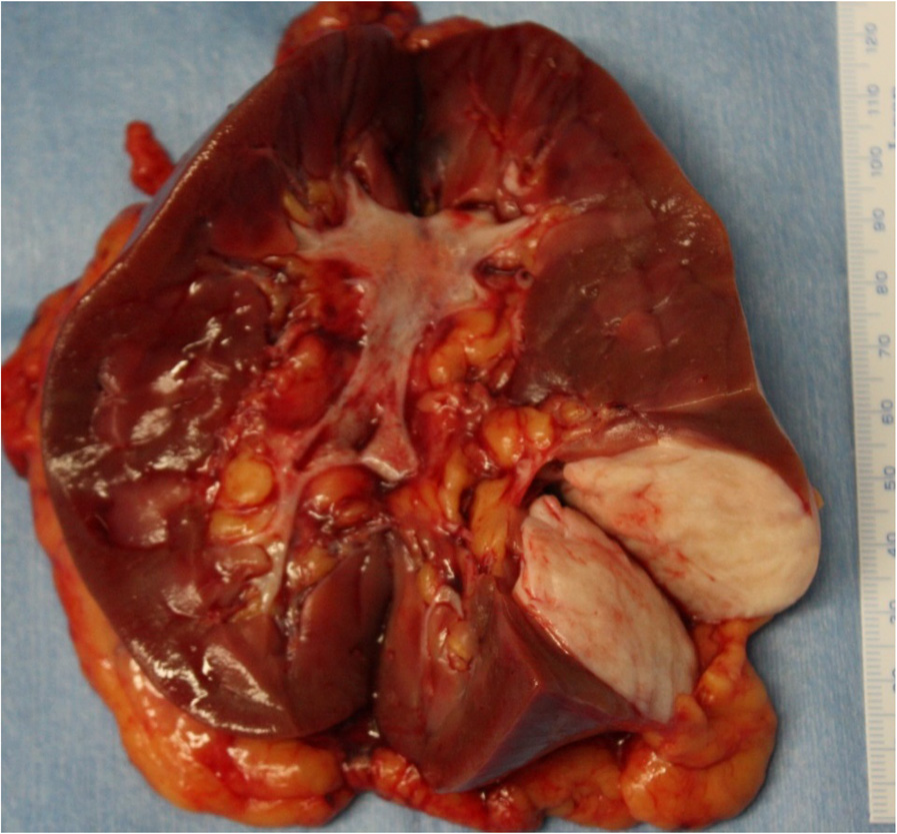
Cut section of the resected kidney and tumor. Upon gross inspection, the tumor located in the lower and dorsal part of the left kidney had expansive growth, was well-demarcated from the renal parenchyma, and had a whitish color. Neither necrosis nor bleeding were observed on the cut surface of the tumor

Macroscopically, the tumor mass, which was buried mostly in the renal parenchyma and displayed endophylic growth, was situated in the lower pole of the left kidney, as shown on USG and CT images. The resected margins of the tumor mass were clear. There was no invasion into the renal capsule, perinephric fat tissues, and the renal parenchyma adjacent to the mass. The excised section of the tumor was gray to white in color and firm upon palpation. Necrosis and hemorrhage were absent on gross inspection (Fig. 2).

**Fig. 3:**
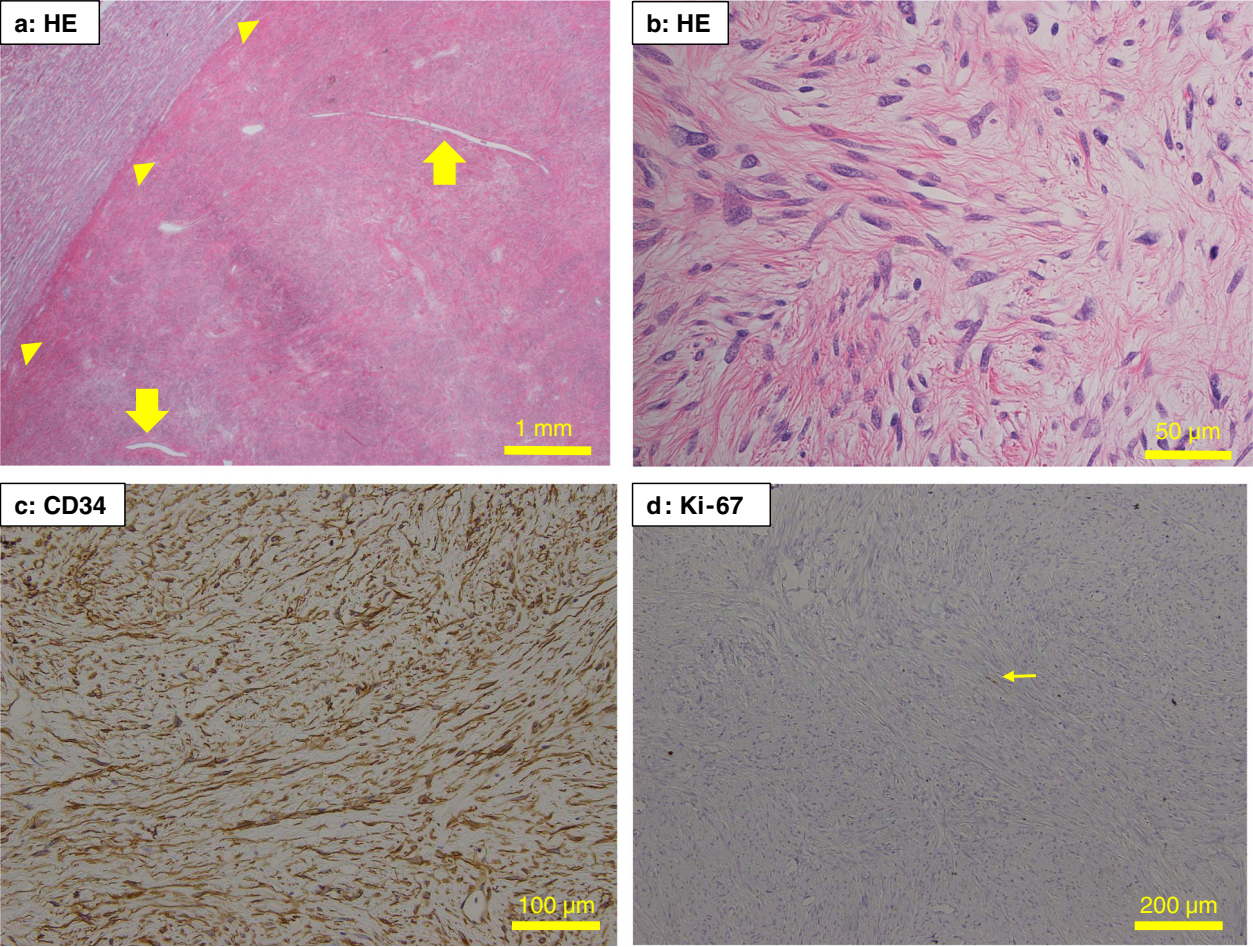
Microscopy of conventional staining of pathological specimens. The tumor had slit-like vessels (**a**, *arrows*) and expansive compression of the adjacent normal renal tissues (**a**, *arrowheads*). The tumor constituted spindle-shaped cells situated in variable directions in dense collagenous stroma (**b**) and had diffuse CD34 expression throughout the tumor (**c**). A small number of tumor nuclei were positive for Ki-67, with a Ki-67 labeling index of 0.6–0.7 % (**d**, *arrow*). Mitosis, atypia, necrosis and hemorrhage were not found by microscopy in the tumor (**a** to **d**). These findings about the kidney tumor are consistent with a classic SFT without malignant nature. Original magnification: x20, x400, x200 and x100 for A, B, C and D, respectively. HE, hematoxylin and eosin

**Fig. 4:**
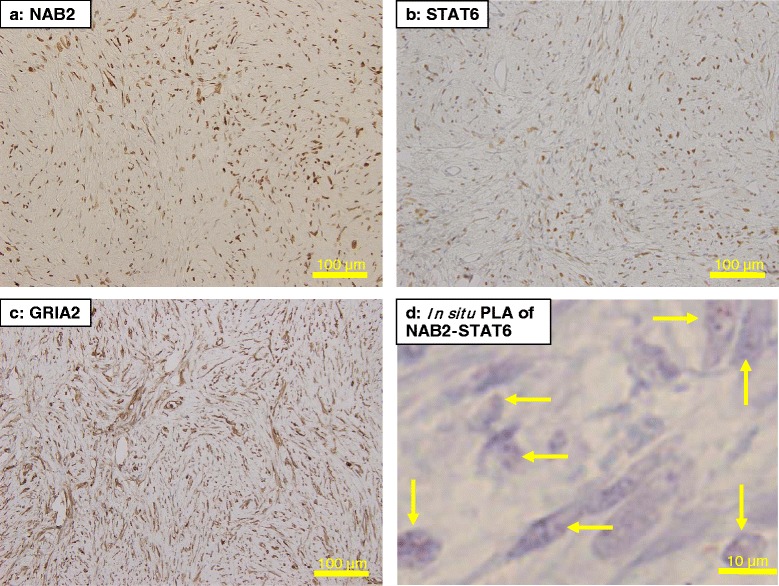
Immunohistochemistry for NAB2 (**a**), STAT6 (**b**), and GRIA2 (**c**). The tumor cells diffusely expressed the antigens. **d** shows the *NAB2-STAT6* gene fusion in the renal SFT cells in an *in situ* proximity ligation brightfield assay, as represented by red dots (*arrows*). PLA, proximity ligation brightfield assay. Original magnification: x200 for **a**, **b** and **c**, and x400 for **d**, respectively

**Fig. 5:**
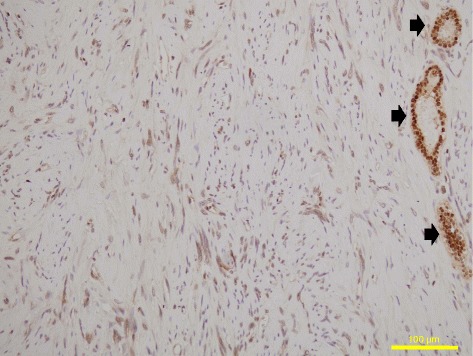
Immunohistochemistry for PAX8. The PAX8 antigen was expressed diffusely in the nuclei of the tumor cells (with spindle-shape nuclei) and the renal tubules (with round nuclei, *arrow*). Original magnification: x200

Microscopically, the tumor was composed of spindle-shape cells distributed variably in the dense collagenous stroma (so-called “patternless pattern”) and had a focal hemangiopericytomatous staghorn-like vascular pattern (Fig. 3a and b). Mitotic figures and atypical structures were not identified. There were no necrotic and hemorrhagic findings in the tumor. In IHC, the tumor cells were negative for desmin, S-100, c-Kit, CK-AE1/AE3, EMA, CD31, CD117, CDK4, MDM2 and p63. The cells were diffusely positive for CD34, CD99, Bcl2, vimentin, α-smooth muscle actin, NAB2, STAT6, and GRIA2 (Figs. 3c and 4) [3, 4, 26, 29]. Ki-67 levels were very low in the tumor nuclei (0.6 to 0.7 %) (Fig. 3d). The microscopic findings were consistent with classic SFT without malignant histology as described by previous studies [7, 19, 8]. PAX8, a marker for renal differentiation, was also positive diffusely in the tumor nuclei (Fig. 5).

Two cases of pleural SFTs with non-malignant nature, obtained from the pathological archives in our institution, were used as positive controls for examining reactivity for various antibodies, including NAB2, STAT6, and GRIA2. One case showed positive staining for both STAT6 and GRAI2, while the other was STAT6-negative but GRIA2-positive (data not shown). The result was similar to a report by Vivero et al. [4]. The former case was positive for NAB2, but the latter was negative (data not shown).

Paraffin-embedded formalin-fixed specimens used for routine pathological investigation were available for IHC of the SFT in the present study. IHC protocols using various primary antibodies are described elsewhere in Additional file 1.

### Identification of a NAB2-STAT6 gene fusion in the SFT

Fresh frozen tumor specimens resected surgically from the patient at the Yamagata University Hospital were used for detection of a *NAB2-STAT6* gene fusion by quantitative reverse transcription-polymerase chain reaction (RT-PCR) and immunoblotting, as reported elsewhere [30, 23, 31, 32]. In addition to the SFT mass, normal renal tissue was compared as a control. PCR primers were designed with reference to previous papers [21, 20, 23]. Structures of the primers used in this study are shown in Additional file 2. *In situ* proximity ligation brightfield assay (PLA) was performed for IHC detection of nuclear *NAB2-STAT6* fusion in the SFT using a Duolink® kit together with anti-NAB2 and -STAT6 primary antibodies. A brief description of the methodology of RT-PCR, direct sequencing and *in situ* PLA in the present study is presented in Additional file 1.

**Fig. 6:**
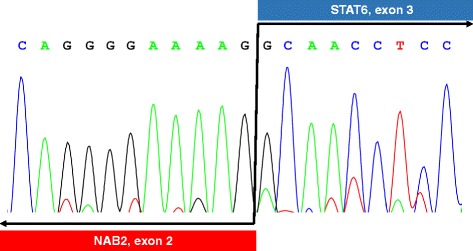
Direct sequencing of the *NAB2-STAT6* gene fusion. The junction of the gene fusion were found at 831 bp within *NAB2* in exon 2 and the beginning of *STAT6* in exon 3

The results of RT-PCR and direct sequencing are presented in Additional file 3 (Additional file 3: Fig. S1) and Fig. 6, respectively. RT-PCR with various combination of forward and reverse PCR primers indicates that a *NAB2-STAT6* gene fusion occurred at some point between exon1 of *NAB2* and exon5 of *STAT6* (Additional file 3). Direct sequencing demonstrated that the fusion junction occurred between *NAB2* in exon2, truncated at 831 kb, and the beginning of *STAT6* in exon3 (Fig. 6 and Additional file 4). In addition, an *NAB2-STAT6* fusion gene was identified as red nuclear dots in an *in situ* PLA assay of pathological sections of the renal SFT (Fig. 4d).

### Discussion

Primary SFTs in the kidney are extremely rare among previous reports of extrathoracic SFTs [8]. Very recently, a growing number of papers on SFT have been published, describing an *NAB2-STAT6* fusion gene in the tumor [20–22, 24, 33], the diagnostic relevance of STAT6 or GRIA2 IHC [4, 5, 25, 26], relationships between fusional variations of the *NAB2-STAT6* gene, histological features and clinical prognosis [23, 24, 30], and expression profiles of genes other than *NAB2-STAT6* [24, 34]. However, these reports are based on SFT specimens from case studies that do not include primary renal SFT or do not describe the specific site of isolation. Diffuse positivity of PAX8 in the nuclei of the present SFT may reflect its renal origin (Fig. 5).

Originally introduced by England et al. [19], the diagnostic criteria for malignant SFT are increased cellularity, pleomorphism, necrosis and hemorrhage, and mitotic activities > 4 counts/10 HPFs on microscopy [8, 7, 19]. In a retrospective analysis of 83 SFT patients, including 59 extrathoracic SFTs, who underwent surgical resection, Wilky et al. reported that malignant histology was very strongly associated with recurrence, but that extrathoracic location could also independently predict recurrence [35]. Therefore, the renal SFT in the present case may have malignant potential, although it is considered benign, both microscopically and clinically, as there has been no distant metastasis or invasion into adjacent tissues in a radical nephrectomy, and no recurrence since the operation. Thus, there is generally no strict correlation between histology and clinical behavior in SFT. It would be more appropriate to describe SFT without malignant nature in histological and clinical presentation as “classic” SFT rather than “benign” SFT.

Renal SFT is presumed to originate from s renal capsule, interstitial or peripelvic connective tissue [7, 8]. Reported in a review [8], the renal capsule is the most common site of the origins. The renal SFT in the present report may occur in intrarenal interstitial tissue because it displayed mostly endophylic growth within the kidney.

Recently, Barthelmeß et al. [23] demonstrated that different patterns of the *NAB2-STAT6* gene fusion could cause specific histology and distinct clinical behaviors in SFT. In their report, twelve different *NAB2-STAT6* fusion variants were identified in 48 of 52 study patients. These were classified by microscopy into three groups, *NAB2* exon4-*STAT6* exon2/3 (n = 27), *NAB2* exon6-*STAT6* exon16/17 (n = 11), and other patterns (n = 10). The first group corresponded to classic thoracic SFT with diffuse fibrosis and non-malignant nature. The second group represented SFT from deep soft tissue with aggressive behavior and poor prognosis. The recurrence rates in the two groups were 15 % and 64 %, respectively (p < 0.016) [23]. With a *NAB2* exon2 (internal)-*STAT6* exon3 fusion, the renal SFT in our case was consistent with Barthelmeβ et al.’s third group of other variants of *NAB2-STAT6* gene fusions. On average, the third group had mitotic counts of 1.0/10 HPFs, a tumor diameter of 3.7 cm and classic characters for SFT in microscopic examination. However, it had a clinical recurrence rate of 10 % over a mean postoperative follow-up of 7.8 years [23]. Late recurrence ≥10 years after initial diagnosis can occur in SFT and some cases can behave aggressively even in the absence of any primary morphologic evidence of malignancy [36]. In our case, only two recurrence-free years with regular medical follow-up have elapsed since radical nephrectomy. Therefore, long-term follow-up should be recommended because the probability of future tumor recurrence in our patient cannot be definitively excluded. On the other hand, careful attention must be paid when directly extrapolating their results for the renal SFT in presented here. One reason caution is required is that their findings were based on the analysis of SFT samples with non-renal primary origins. Secondly, *NAB2-STAT6* gene fusions were not necessarily detected in about 10–50 % of patients with SFT [21, 22, 24, 33], implicating that abnormal fusion of the *NAB2-STAT6* gene is not be essential and sufficient for oncogenesis in SFTs. Currently, variations of the *NAB2-STAT6* gene fusion cannot explain clear relationships in tumorigenesis, the preponderance of the thoracic origin, or the malignant behavior of SFT.

Differentiation and definitive diagnosis of SFT from other soft tissue tumors has been regarded as difficult due to histological similarities [4]. IHC against CD34, CD99, and Bcl-2 are often used as supportive diagnostic stains for SFT, and 90–95 %, 70 %, and 20–35 % of SFT cases are positive, respectively [8, 5]. However, other soft tissue tumors are also frequently positive for CD34, CD99, and Bcl-2, rendering these antigens non-specific for SFT [4]. Dedifferentiated liposarcoma, which shows the similar morphology to SFT on microscopy, was differentiated because IHC for MDM2 and CDK4 was negative in the present case [37]. Analysis of IHC on a large number of sarcomas reported a sensitivity of 95 % and 92 % and a specificity of 81 % and 95 % for, respectively, MDM2 and CDK4 for the diagnosis of dedifferentiated liposarcoma [37]. Based on evidence that a *NAB2-STAT6* gene fusion is present in most SFT cases, NAB2 and STAT6 IHC have recently been reported in 100 % [24, 33] and 86–100 % [23–26, 29] of SFT cases. Surprisingly, only 2.3–2.5 % of non-SFT soft tissue tumors exhibited positive and weak reactions in IHC with anti-STAT6 antibodies [25, 26]. Thus, STAT6 IHC could be a valuable diagnostic standard for the NAB2-STAT6 fusion protein in SFT [5, 26, 25]. Vivero et al. demonstrated that GRIA2 was a useful marker for distinguishing SFT from most mimics, as 89 % of SFTs were GRIA2-positive, while only 5–10 % of other soft tissue tumors were positive, except for dermatofibrosarcoma and myoepithelioma [4]. In their report, a noteworthy case of SFT proven to have the *NAB2-STAT6* gene fusion was STAT6-negative but GRIA2-positive in IHC.

In normal cells, *NAB2* and *STAT6* are located close together on chromosome 12 and transcribed in opposite directions [26]. STAT6 and NAB2 function antagonistically as a transcription activator and a repressor, respectively, regulating wound healing and fibrosis via early growth response 1-mediated pathways [26]. GRIA2, a glutamate receptor subunit, affects cell membrane calcium permeability, cell proliferation, motility, and cell death [4]. GRIA2 is normally expressed in the central nervous system under physiological conditions, but GRIA2 can be detected in various oncogenic conditions via unknown mechanisms [4, 22]. In the present study, the renal SFT, which had a *NAB2-STAT6* gene fusion identified by direct sequencing, was stained positively with anti-NAB2, -STAT6, and -GRIA2 antibodies in IHC. However, one pleural SFT, used as a positive IHC control in the present analysis, was negative for NAB2 and STAT6 in IHC but positive for GRIA2. This finding of reciprocal staining of STAT6 and GRIA2 in SFT may lead the future identification of an alternative pathway of tumorigenesis in SFT. Our findings are based on a renal SFT and two pleural SFT cases. Similar to remarks from Kuroda et al. [7], we also think that a broader investigation will be necessary to clarify the etiology of SFT and discuss its clinical characteristics and prognosis.

## Conclusions

For the first time, we have reported a case of GRIA2-positive SFT occurring primarily in the kidney with a *NAB2* (exon 2, internal)-*STAT6* (exon 3) gene fusion for the first time. It might present with tumor background of a renal origin that the tumor cells in the present SFT expressed diffusely not only STAT6, NAB2 and GRIA2 but also PAX8. Indeed, clinical prognosis cannot be necessarily predicted by classic/non-malignant histology of SFT, but analysis of genetic alterations in SFT would help to ameliorate prognostic prediction of the tumor. In the present case, long-term follow-up should be performed after radical nephrectomy, as classic SFT from an extrathoracic origin may have malignant potential.

## Consent

Written informed consent was obtained from the patient for publication of this Case Report, any accompanying images, clinical data, and results of the tumor gene analysis. A copy of the written consent is available for review from the Editor-in-Chief of this journal.

The present study was performed in accordance with the principles embodied in the Declaration of Helsinki and approved by the Ethical Committee of Yamagata University Faculty of Medicine (approval No.6, 2015).

## Additional files

Additional file 1:
**Methods for immunohistochemistry, identification of** ***NAB2-STAT6*** **gene fusion and in situ proximity ligation brightfield assay.** (PDF 67 kb) Additional file 2:
**Primers used for reverse transcription-polymerase chain reaction and direct DNA sequencing.** (PDF 31 kb) Additional file 3:
**Results of reverse transcription-polymerase chain reaction (RT-PCR).** Fusion of the *NAB2-STAT6* gene was detected by RT-PCR with several sets of specifically designed forward and reverse PCR primers. Sequences of the PCR primers are given in Additional file 2. SFT, solitary fibrous tumor. (PNG 73 kb) Additional file 4:
**Summary of the** ***NAB2-STAT6*** **gene fusion and PCR primers used in the present study.** (PNG 64 kb)

## Abbreviation

CT: Computed tomography
GRIA2: Glutamate receptor, inotropic, AMPA 2
IHC: Immunohistochemistry
NAB2: NGFI-A binding protein 2
RCC: Renal cell carcinoma
RT-PCR: Reverse transcription-polymerase chain reaction
SFT: Solitary fibrous tumor
STAT6: Signal transducer and activator of transcription 6
USG: Ultrasonography
