# Supplementary material for: A GRIA2 and PAX8-positive renal solitary fibrous tumor with NAB2-STAT6 gene fusion
Source: Diagn Pathol. 2015 Sep 4;10:155. doi: 10.1186/s13000-015-0386-x (PMC4559176; doi:10.1186/s13000-015-0386-x)
Supplement: Additional file 1: — Methods for immunohistochemistry, identification of NAB2-STAT6 gene fusion and in situ proximity ligation brightfield assay. (PDF 67 kb) [file 13000_2015_386_MOESM1_ESM.pdf]

Additional File1

Methods

Immunohistochemical staining

Immunohistochemistry (IHC) for desmin, S-100, CK-AE1/AE3, CD34, CD99, Bcl2, vimentin, α-smooth muscle actin (α-SMA), Ki-67 (all from DAKO Japan, Tokyo, Japan), STAT6 (sc-621; Santa Cruz Biotechnology, Santa Cruz, CA, 1:1000 dilution), NAB2 (sc-23867, Santa Cruz Biotechnology, 1:100 dilution), GRIA2 (ab52896; Abcam, Tokyo, Japan, 1:100 dilution) and PAX8 (10336-1-AP; ProteinTech Group, Chicago, IL, 1:50 dilution) were carried out with a standard procedure, as described elsewhere [25, 32, 36, 37]. Briefly, tissue sections (3μm) were deparaffinized in xylene, rehydrated. Endogenous peroxidase was blocked with 3% H_2O_2 in methanol for 15min. Sections were incubated with primary antibodies overnight after heat-induced antigen retrieval with a pressure cooker (120°C, 10min). Detection was performed by the peroxidase method using 3,3'-diaminobenzidine in the presence of H_2O_2. The sections were counterstained with haematoxylin for nuclear detection, dehydrated, and mounted as permanent histological specimens. Appropriate positive and negative controls were used for IHC in

the present study.

Identification of a NAB2–STAT6 *gene fusion in the SFT sample*

Reverse transcription-polymerase chain reaction (RT-PCR) and direct sequencing were used to evaluate the *NAB2-STAT6* gene fusion. Briefly, total RNA was extracted from fresh frozen samples using a mirVana miRNA Isolation Kit (Life Technologies Japan, Tokyo, Japan). The first-strand complementary DNA was synthesized from the RNA extracts with a High-Capacity cDNA Reverse Transcription Kit (Life Technologies Japan, Tokyo, Japan).

The *NAB2–STAT6* gene fusion was examined via RT-PCR with specifically designed sets of PCR primers, with reference to previous papers [20, 21, 23]. Direct sequence analysis of the PCR products was performed using the Big-Dye terminator method (version 1.1; Applied Biosystems, Foster City, CA) to confirm the breakpoints of fusion transcripts [20, 21, 23] [32]. Primers for RT-PCR and direct DNA sequencing are shown in Table 1.

In situ *proximity ligation brightfield assay*

An *in situ* proximity ligation brightfield assay for IHC of nuclear *NAB2-STAT6* fusion in SFT was performed using primary antibodies against NAB2 (monoclonal mouse, clone 1C4: sc-23867, Santa Cruz Biotechnology, Santa Cruz, CA, 1:50 dilution) and SATA6 (polyclonal rabbit, clone S-20: sc-621; Santa Cruz Biotechnology, 1:50 dilution), according to previous studies [5, 33] and the manufacturer's instruction (Duolink® In Situ Detection Reagents Brightfield, Duo92012; Sigma-Aldorich Japan, Tokyo, Japan). Briefly, 3μm sections of paraffin blocks were pretreated in citrate buffer (pH 6) in an autoclave at 100°C for 20 min. Intrinsic peroxidase quenching and blocking was performed with the vender's reagent (Duo92012). Slides were incubated with primary anti-NAB2 and anti-STAT6 antibodies at 4°C overnight. Subsequent protocols, such as incubation with secondary antibodies, ligation, amplification, detection reaction, and nuclear counterstaining, were performed using the Duolink® kit according to the manufacturer's instructions [5, 33].
