# Supplementary material for: A GRIA2 and PAX8-positive renal solitary fibrous tumor with NAB2-STAT6 gene fusion
Source: Diagn Pathol. 2015 Sep 4;10:155. doi: 10.1186/s13000-015-0386-x (PMC4559176; doi:10.1186/s13000-015-0386-x)
Supplement: Additional file 2: — Primers used for reverse transcription-polymerase chain reaction and direct DNA sequencing. (PDF 31 kb) [file 13000_2015_386_MOESM2_ESM.pdf]

Table 1. Primers used for reverse transcription-polymerase chain reaction and direct DNA sequencing

Primers for RT-PCR	Name	Sequence
Forward (5'-3')	F_NAB2ex1A	aggctcggagagagaagacg
	F_NAB2ex1C	ggacagagcctggacagc
Reverse (5'-3')	R_STAT6ex5	gaaagtggccaccagcttca
	R_STAT6ex8	ctctagctctccagtggt
	R_STAT6ex11	cagttcccaggaatgctgtt
	R_STAT6ex15	caaaccactgccaaaaggtg
	R_STAT6ex18	gatggtagctgggacataacc
	R_STAT6ex23	agtgaggtcctgttcagtgg

Primer for direct DNA sequencing	Name	Sequence
Forward (5'-3')	F_NAB2ex2B	tctggagatcatggcacttgt
	F_NAB2ex2C	atccagggctcttcagtcaa
Reverse (5'-3')	R_STAT6ex3	aagatgcccccagaaaaagt
	R_STAT6ex5	gaaagtggccaccagcttca

RT-PCR: reverse transcription-polymerase chain reaction
