# Supplementary figures and images for: A GRIA2 and PAX8-positive renal solitary fibrous tumor with NAB2-STAT6 gene fusion
Source: Diagn Pathol. 2015 Sep 4;10:155. doi: 10.1186/s13000-015-0386-x (PMC4559176; doi:10.1186/s13000-015-0386-x)

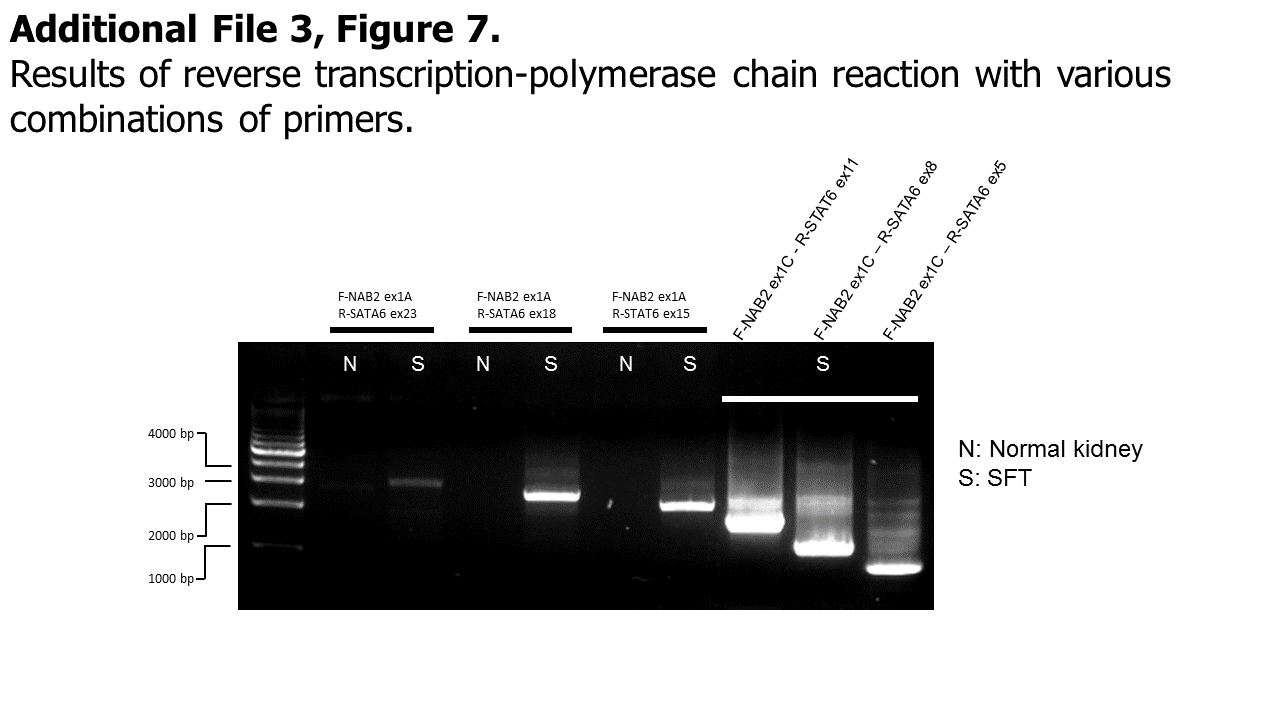

Supplement: Additional file 3: — Results of reverse transcription-polymerase chain reaction (RT-PCR). Fusion of the NAB2-STAT6 gene was detected by RT-PCR with several sets of specifically designed forward and reverse PCR primers. Sequences of the PCR primers are given in Additional file 2. SFT, solitary fibrous tumor. (PNG 73 kb) [file 13000_2015_386_MOESM3_ESM.png]

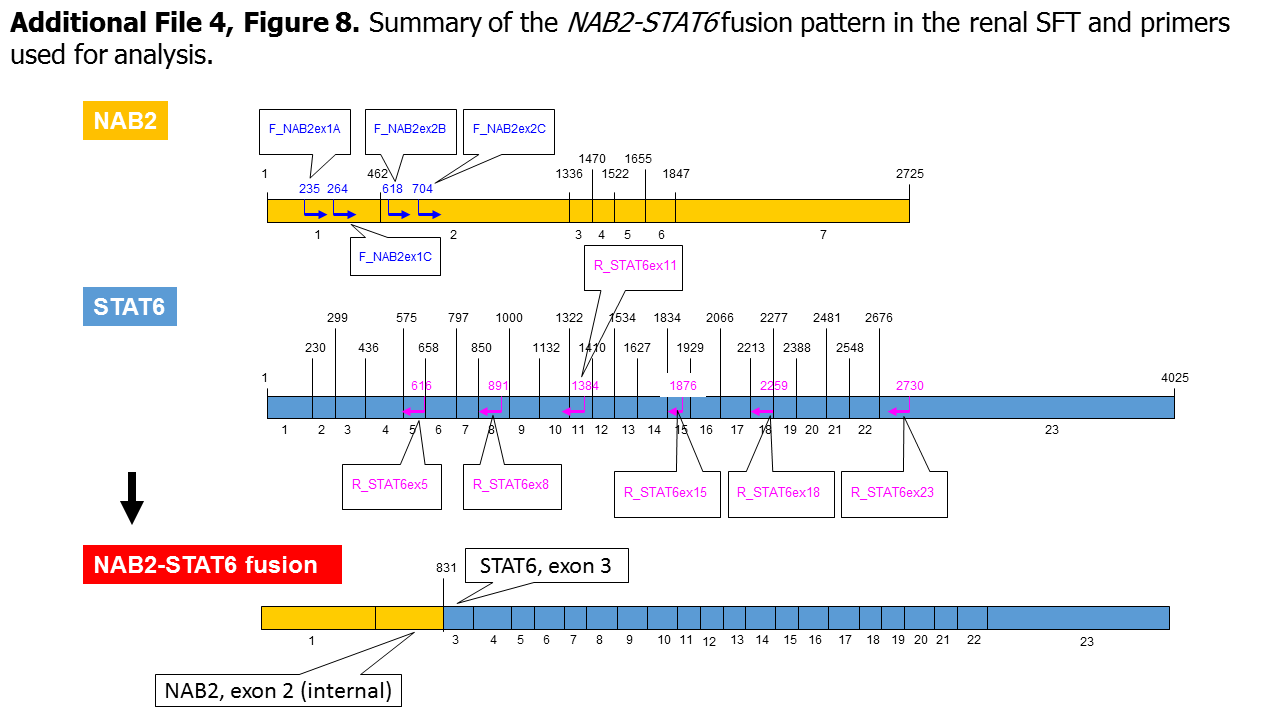

Supplement: Additional file 4: — Summary of the NAB2-STAT6 gene fusion and PCR primers used in the present study. (PNG 64 kb) [file 13000_2015_386_MOESM4_ESM.png]
